# Temperature Effects on Development and Population Growth of Two Parasitoids (Hymenoptera) of the Coffee Berry Borer (*Hypothenemus hampei*)

**DOI:** 10.1007/s13744-025-01356-5

**Published:** 2026-02-17

**Authors:** Marisol Giraldo-Jaramillo, Melissa A. Johnson, Peter Follett, Pablo Benavides

**Affiliations:** 1 Department of Entomology, National Coffee Research Center, Cenicafé, Manizales, Colombia; 2 United States Department of Agriculture – Agricultural Research Service, Daniel K. Inouye U.S. Pacific Basin Agricultural Research Center, Hilo, HI USA

**Keywords:** Biological control, *Coffea arabica*, Developmental threshold, Fecundity, Longevity, Sex ratio

## Abstract

Coffee berry borer, *Hypothenemus hampei* Ferrari (Coleoptera: Curculionidae), is the most damaging pest of coffee worldwide, reducing both yields and quality. African parasitoid wasps have been widely released in Colombia as biological control agents for *H. hampei*, yet their establishment has been inconsistent, partly due to limited information on how temperature affects their performance. We evaluated the thermal biology of two key parasitoids of *H. hampei*: the larval-pupal ectoparasitoid *Prorops nasuta* Waterston (Hymenoptera: Bethylidae) and the adult endoparasitoid *Phymastichus coffea* LaSalle (Hymenoptera: Eulophidae). Using age-stage, two-sex life tables across eight constant temperatures, we quantified temperature effects on development, survival, fecundity, and population growth. We identified 22–25 °C as the optimal range for survival, and fecundity peaked at 25 °C. Life-table parameters indicate maximal population growth at 25 °C for both parasitoids. Development failed at extreme temperatures (32 and 35 °C), highlighting upper thermal limits relevant to mass-rearing and field releases for both parasitoids. Degree-day models were developed to estimate the potential number of generations across Colombian coffee-growing regions. We predict 5.5–11.6 annual generations of *P. nasuta* and 2.2–8.6 of *P. coffea*, depending on local temperature regimes. These results identify optimal temperature ranges for rearing and deploying *P. nasuta* and *P. coffea* and provide spatially relevant predications for their establishment potential in Colombian coffee-growing regions. Regions with mean temperatures between 22 and 28 °C are expected to support the greatest efficacy of augmentative biological control programs targeting the coffee berry borer.

## Introduction

Coffee berry borer (CBB, *Hypothenemus hampei* Ferrari) (Coleoptera: Curculionidae) is the most damaging insect pest of coffee, resulting in more than half a billion dollars in annual losses worldwide (Vega et al. 2015). The adult female CBB bores a hole into the coffee fruit and then enters the endosperm (bean), digging tunnels for reproduction. The larvae feed on the endosperm tissue, causing further damage to the bean and reducing both yields and quality (Duque and Baker 2003). Various IPM strategies have been used by different countries to mitigate the economic impact of this insect, including monitoring, insecticide sprays, sanitation, and biological control (Aristizábal et al. 2016, 2023; Infante 2018; Johnson et al. 2020). CBB has many natural enemies in its native Africa, including parasitoid wasps (Escobar-Ramirez et al. 2019) that act as biological controls for this pest. While biological control is one of the most environmentally friendly and sustainable components of IPM strategies for CBB, success is often limited by challenges in mass-rearing, including humidity sensitivity during development, difficulties synchronizing host availability, and low adult survival during handling and transport (Infante et al. 2001a; Jaramillo et al. 2006; Vega et al. 2015; Aristizábal et al. 2016; Escobar-Ramirez et al. 2019).

Biological control of *H. hampei* has been conducted in many countries around the world, especially in Latin America (Mexico south to Brazil) (Barrera et al. 1990; Infante et al. 2001). The African parasitoids of *H. hampei* that have been successfully reared in the laboratory and used in inoculative and augmentative biological control programs in Latin America include two ectoparasitic bethylid wasps, *Cephalonomia stephanoderis* Betrem (Hymenoptera: Bethylidae) and *Prorops nasuta* Waterston (Hymenoptera: Bethylidae), that attack larvae, and one endoparasitic eulophid wasp, *Phymastichus coffea* LaSalle (Hymenoptera: Eulophidae), that attacks adults. In Colombia, recent augmentation biological control (i.e., the mass-rearing and release of non-native enemies for pest control) has focused on *Prorops nasuta* and *Phymastichus coffea*. *Prorops nasuta* has been recorded from all coffee-growing regions in Colombia and from 65% of the coffee farms located between 1150 and 1840 m elevation (Maldonado and Benavides 2007). Benavides et al. (2023) conducted a two-year large-scale field study of augmentation biological control against *H. hampei* using *P. nasuta* and *P. coffea*. Multiple releases of *P. nasuta* reduced *H. hampei* populations by as much as 81%. Parasitism rates of *P. coffea* reached 51–77%, with higher parasitism in warm, dry areas. However, despite high parasitism, establishment was poor, and *P. coffea* populations typically disappeared within five months after release (Benavides et al. 2023).

Insect development, behavior, feeding, survival, and reproduction are all highly influenced by climatic conditions, especially temperature (Chapman 1998; Infante 2000). The use of parasitoids as biological control agents for *H. hampei* may thus be improved by using information on the effect of temperature on various life-history characteristics, thereby optimizing rearing and release conditions (Infante et al. 2005). Life tables are a useful tool for quantifying the effects of temperature on insect population dynamics (Gao et al. 2012; Fonseca-Lacerda et al. 2019).

This study evaluated the age-stage life table parameters of *P. nasuta* and *P. coffea* at eight constant temperatures in the laboratory. We also estimated the number of *P. nasuta* and *P. coffea* generations per year in the Colombian coffee-growing region to predict where the environment will be most favorable for parasitoid establishment so that suitable areas can be targeted for future field releases. Determining optimal rearing temperatures will also improve augmentation biological control using mass-reared parasitoids and hosts. A better understanding of thermal biology and more accurate estimates of lower and upper developmental thresholds, thermal constants, and optimal temperatures for development should allow for improved efficacy of biological control in similar climates.

## Material and Methods

### Study Species

*Prorops nasuta* is a solitary idiobiont ectoparasitoid wasp that originated in East Africa (Hempel 1934). This species is highly host-specific and attacks all life stages of *H. hampei*, acting as both a predator and parasitoid of *H. hampei* (Bacca 1999). When the female *P. nasuta* wasp enters a *H. hampei*-infested coffee fruit, it first kills the adult *H. hampei* and blocks the entrance to the berry with the *H. hampei* mummy (Hempel 1934; Infante et al. 2005). Over a period of 3–14 days, the wasp will feed on the immature stages of *H. hampei* and paralyze the full-grown larvae and pupae (Lauzière et al. 2001; Infante et al. 2005). Eggs are laid externally on the last instar larvae and pupae of *H. hampei* (Infante et al. 2005). After incubation, the *P. nasuta* larvae feed on a single host immediately after hatching and continue their development (Infante et al. 2005). Females emerge from their pupal case 2.5 days after males (Infante et al. 2005). Female bethylid wasps enter only one *H. hampei*-infested berry during their life and attack the *H. hampei* life stages within.

*Phymastichus coffea* is a gregarious idiobiont endoparasitoid wasp that is native to West Africa and is highly host-specific (Yousuf et al. 2021). Given that *P. coffea* is the only known parasitoid of adult *H. hampei*, several studies have suggested that *P. coffea* could be complementary to the action of bethylid parasitoids in controlling *H. hampei* (Lopez-Vaamonde and Moore 1998; Baker 1999; Gutierrez et al. 1998). *Phymastichus coffea* attacks the adult female *H. hampei* while it is colonizing the coffee berry, thereby preventing damage to the seed (“bean”). The adult female *P. coffea* typically lays two eggs per *H. hampei* adult; the female larva feeds and develops in the abdomen while the smaller male larva migrates to the prothorax. The male and female presumably mate within the host just before the female makes an exit through the tip of the *H. hampei* abdomen (Espinoza et al. 2009). Adult *Phymastichus coffea* females can attack ~ 10 *H. hampei* adults during their lifespan (Orozco Hoyos 2002; Espinoza et al. 2009).

### Insect Rearing

The study was conducted in the Planta Piloto of the Entomology Department at the National Coffee Research Center (Cenicafé) in Manizales (Caldas), Colombia. *Hypothenemus hampei* individuals were obtained from laboratory colonies that originated from infested berries collected in the field. Rearing was done on parchment coffee beans (45% moisture content) following the methods of Gómez et al. (2024) or green coffee-based artificial diet following the methods of Giraldo-Jaramillo and Parra (2018) and kept in the dark at 25 ± 2 °C and 65 ± 10% RH. A colony of *P. nasuta* that originated in Kenya has been maintained under laboratory conditions at Cenicafé, Colombia, since 1989; a colony of *P. coffea* that originated in Togo has been maintained at Cenicafé since 1996 (Bustillo 2006). For this study, a laboratory colony of *P. nasuta* was reared on immature stages of *H. hampei* and a laboratory colony of *P. coffea* was reared on adult *H. hampei*. Both colonies were maintained at 25 ± 2 °C and 65 ± 10% RH. Because immature parasitoids develop inside host tissues within berries and are not exposed to natural light cues, we used continuous darkness for developmental assays (0L:24D).

### Parasitism of ***Hypothenemus hampei*** in the Laboratory

*Prorops nasuta* eggs were obtained according to the methodology described by Infante et al. (1992), with slight modifications. To initiate the experiment with *P. nasuta*, Petri dishes were set up with 60 mL of artificial diet and the host *H. hampei*. Within each Petri dish, 100 *H. hampei* larvae and 100 *H. hampei* pre-pupae were placed on the surface of the diet. Twenty *P. nasuta* wasps were then released per day in each of the 20 Petri dishes for five consecutive days. Each day, the parasitized immature *H. hampei* (those that were observed to have eggs laid on them) were removed and transferred to a new Petri dish with artificial diet. Petri dishes with parasitized insects were placed in one of eight environmental chambers, each set to a different constant temperature (16, 19, 22, 25, 28, 30, 32, or 35 °C). Therefore, 20 replicate Petri dishes with parasitized *H. hampei* were included for each temperature treatment. The RH in all chambers remained set to 65 ± 10% RH, while light conditions were transitioned to a 12L:12D photoperiod during emergence and parasitism to reflect natural light conditions experienced by adults and to support host-seeking and reproductive behavior. To initiate the experiment with *P. coffea*, adult *H. hampei* were placed on a thin layer of artificial diet and exposed to newly emerged adult *P. coffea* females according to the methodology developed at Cenicafe (Gómez et al. 2024). The ratio of *P. coffea* to *H. hampei* was 1:10 (Orozco Hoyos 2002). Following exposure, the parasitized *H. hampei* were collected from the artificial diet and assigned to cohorts of 3,000 individuals to be placed in each of the eight temperature treatments.

### Development, Survival, and Sex Ratio

For each temperature, the development of 200 *P. nasuta* individuals was examined daily from egg to adult emergence. Although 3000 parasitized adults were placed in each temperature treatment for *P. coffea*, only 50 individuals per day were randomly selected for dissection. This subsampling approach is standard for *P. coffea*, whose gregarious development yields sufficient statistical power without monitoring all individuals. The duration of each life stage (egg, larva, pupa, and adult) and stage-specific survival was recorded until all the adults had emerged. Parasitoid sex ratio was determined by counting males and females that emerged from the undissected parasitized *H. hampei* at each temperature.

### Longevity and Fecundity

To determine the longevity of *P. nasuta* wasps and the total number of eggs per female (gross fecundity) at each temperature, adults were placed into pairs (1 female + 1 male) approximately 6 h after emergence. Each pair was placed in a borosilicate glass vial (0.9 cm in diameter and 3.4 cm in height, covered with a plastic top perforated with a 1-mm opening), containing one parchment coffee bean that had been infested with two *H. hampei* females 20 days prior and had an average of 40 ± 5 immature *H. hampei* per bean. Although the number of immature *H. hampei* per bean varied slightly, beans were randomly assigned to treatments, ensuring that any variation was evenly distributed and not systematically associated with temperature. Environmental chambers were set for each of the eight temperature treatments, all of which were programmed to 65 ± 10% RH. In contrast to developmental assays, longevity assays were conducted under a 12L:12D photoperiod to reflect natural adult activity patterns and facilitate mating and oviposition. A total of 100 *P. nasuta* pairs were monitored for each temperature. Every 15 days, all the beans were dissected and the surviving wasps were counted, along with the number of *P. nasuta* eggs and/or larvae per bean. The wasps that were still alive were placed on new parchment coffee infested with two *H. hampei* females as described above. The experiment ended when the last wasp was recorded as dead for each temperature.

Six hours after emergence of adult *P. coffea*, 50 adult female/male *P. coffea* pairs were placed within vials containing *H. hampei* to determine the total number of eggs produced per *P. coffea* female (gross fecundity) and days lived (adult longevity) for each temperature treatment. The vials consisted of borosilicate glass, with dimensions of 0.9 cm in diameter and 3.4 cm high and covered with plastic tape with a 1-mm perforation. Each vial with a mated pair (the experimental unit) contained 30 adult *H. hampei* collected from the laboratory colony and a single parchment coffee bean. Each temperature treatment had 50 pairs of wasps (50 vials) and a total of 1500 *H. hampei*. Wasp survival was recorded daily to estimate longevity at each temperature. Once the female *P. coffea* was found dead, each of the 30 *H. hampei* was removed to determine the number of parasitized *H. hampei*. In addition, the number of eggs per parasitized *H. hampei* was counted and used to determine female gross fecundity at each temperature.

### Estimating Development Rate and Construction of Mathematical Models

The results for the effect of temperature on the *P. nasuta* and *P. coffea* life cycles were used to estimate the development thresholds and thermal constants (Haddad and Parra 1984), and to calculate the development rate, expressed as the reciprocal of the development time in days (1/*d*) of each life stage. The development rate of *P. nasuta* and *P. coffea* immature stages (egg, larva, and pupa) was regressed against temperature using the following linear equation:

1 $$1/d=a+bT$$

where *d* is the development rate (days), and *T* is the temperature (°C). The lower threshold temperature (*t*) was calculated as the ratio of the intercept over the slope of the regression line. The thermal constant (*K*), i.e., the number of degree-days (DD) for egg-to-adult development, was calculated as the reciprocal of the slope (1/*b*) from the regression equation (Haddad and Parra 1984).

### Statistical Analysis

All data were tested for homogeneity (Bartlett test) and normality (Shapiro–Wilk test) with the *stats* package in R v. 4.4.1 (R Core Team 2024). Given that the variances were not equal and the data were non-normal, separate Kruskal–Wallis tests were conducted using the *stats* package to test the effect of temperature on development times, fecundity, longevity, and sex ratio. In the case of significant differences among temperatures, Dunn’s test was applied (*α* = 0.05) as a post hoc for pairwise multiple comparisons using the *dunn.test* package v. 1.3.6 (Dinno 2017). The relationship between development time and temperature at each life stage was described using linear regression with the lm function in the R *stats* package. The performance of the fit of the linear model to the data was described using the coefficient of determination (*R*^2^). We used a generalized linear model (GLM) with a binomial error distribution and the logit-link function in the *stats* package to determine the effect of temperature on survival. The package *emmeans* v. 1.10.6 (Lenth 2024) was used to conduct a follow-on Tukey’s multiple comparison test.

The life table measurements for *P. nasuta* and *P. coffea* were calculated using the age-specific fecundity, number of eggs produced per female, age-specific population size, age-specific development, and age-specific survival rate. We used the TWOSEX-MSChart software (Chi et al. 2023) to calculate the following parameters: *R*_*0*_ = net reproductive rate, *T* = duration of each generation, *r*_*m*_ = intrinsic rate of increase, *λ* = finite rate of increase; and *D*_*t*_ = generation doubling time. Statistical comparison of values at different temperatures was performed using the Bootstrap test in the TWOSEX-MSChart software (Chi and Liu 1985; Chi et al. 2023).

### Estimating Number of Generations

Degree-days were calculated using monthly and annual mean temperatures from the Colombian coffee region. Four different temperature ranges or isotherms (≤ 17 °C, > 17 and ≤ 20 °C, > 20 and ≤ 23 °C, and > 23 °C) were obtained from the Colombian coffee climate network (Sarmiento-Herrera et al. 2022), and the number of *P. nasuta* and *P. coffea* generations was then calculated using the following equation (Arnold 1959):

2 $$NG= \frac{\left(Tm-t\right)*d}{K}$$

where *NG* = number of generations, *Tm* = mean monthly/annual temperature for four isotherms of the Colombian coffee region, *t* = minimum threshold temperature, *K* = thermal constant, and *d* = number of days per month. The ArcMap application in ArcGIS v. 10.3 was used to organize and represent the number of generations in shapefiles. The four different temperature ranges or isotherms (≤ 17 °C, > 17 and ≤ 20 °C, > 20 and ≤ 23 °C, > 23 °C) were assigned different shades of gray to define zones according to the number of generations of *P. nasuta* and *P. coffea*.

## Results

### Development, Survival, and Sex Ratio

**Fig. 1:**
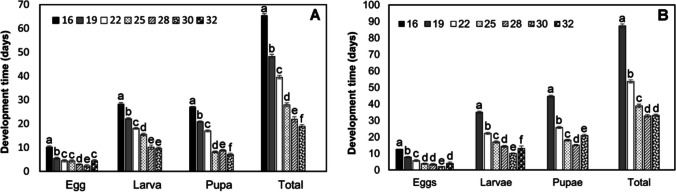
Mean development time (± SD) for life stages of *Prorops nasuta* (**A**) and *Phymastichus coffea* (**B**) at constant temperatures. Different lowercase letters indicate significant differences within a given life stage according to Dunn’s test (*α* = 0.05)

*Prorops nasuta* completed development from 16 to 30 °C (Fig. 1A). Significant differences in development times were observed among the temperatures examined for each of the life stages (eggs: *χ*^2^ = 880.21, df = 6, *p* < 0.001; larvae: *χ*^2^ = 355.26, df = 5, *p* < 0.001; pupae: *χ*^2^ = 726.10, df = 5, *p* < 0.001; total: *χ*^2^ = 636.74, df = 5, *p* < 0.001) (Fig. 1A). The total duration of development was longest at 16 °C (64.8 ± 0.9 days) and shortest at 30 °C (19 ± 0.6 days). Development at 32 °C did not progress past the egg stage, and at 35 °C all *P. nasuta* died at the egg stage (Fig. 1A).

*Phymastichus coffea* completed development at temperatures from 19 to 30 °C (Fig. 1B). Significant differences in development times were observed among the temperatures examined for each of the life stages (eggs: *χ*^*2*^ = 651.64, df = 6, *p* < 0.001; larvae: *χ*^2^ = 573.83, df = 5, *p* < 0.001; pupae: *χ*^2^ = 486.05, df = 4, *p* < 0.001; total: *χ*^2^ = 461.69, df = 4, *p* < 0.001) (Fig. 1B). Development time from egg to adult was the longest at 19 °C (87.35 ± 0.11 days) (mean ± SE) and shortest at 28 °C (32.78 ± 0.08 days) (Fig. 1B). Development at 16 °C did not progress past the egg stage, and at 32 °C did not progress past the larval stage. At 35 °C, all *P. coffea* died at the egg stage (Fig. 1B).

**Fig. 2:**
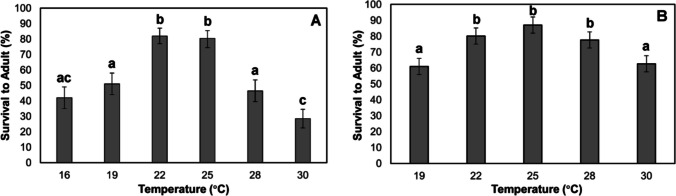
Mean survival (± 95% CI) of *Prorops nasuta* (**A**) and *Phymastichus coffea* (**B**) from egg to adult at constant temperatures. Different lowercase letters indicate significant differences according to Dunn’s test (*α* = 0.05)

Survival from egg to adult in *P. nasuta* was less than 50% at 16 °C, 28 °C, and 30 °C. The optimal temperature range was observed to be 22–25 °C, at which survival from egg to adult was ≥ 80% (Fig. 2A). Lower survival of all stages (egg-adult) was also observed at extreme temperatures for *P. coffea* (Fig. 2B). Only 75% of eggs survived at 16 °C, and no eggs survived at 35 °C. Larval survival was zero at 16 °C and only 41% at 32 °C. Pupal survival was high (≥ 80%) between 19 and 30 °C, with no survival at 32 °C. Adult survival was lowest at 19 and 30 °C (61–63%) and highest at 25 °C (87%) (Fig. 2B).

Temperature significantly affected the sex ratio of *P. nasuta*, with the proportion of females decreasing as temperature increased (Table 1). Results of Dunn’s test showed that the proportion of females was significantly higher at the lowest temperatures of 16 and 19 °C. In contrast, temperature did not affect the sex ratio for *P. coffea*, which was roughly 1:1 at all temperatures (Table 1).

**Table 1:** Proportion of female *Prorops nasuta* and *Phymastichus coffea* observed at six constant temperatures. Different lowercase letters indicate significant differences according to Dunn’s test (*α* = 0.05). Number in parentheses (*n*) indicates total emerged adults per temperature

Temperature (°C)	*Prorops nasuta*	*Phymastichus coffea*
16	0.96 a (n = 200)	-
19	0.83 b (n = 200)	0.51 a (n = 89)
22	0.62 c (n = 200)	0.52 a (n = 129)
25	0.59 c (n = 200)	0.52 a (n = 282)
28	0.36 c (n = 200)	0.52 a (n = 115)
30	0.21 c (n = 200)	0.52 a (n = 74)

### Longevity and Fecundity

**Fig. 3:**
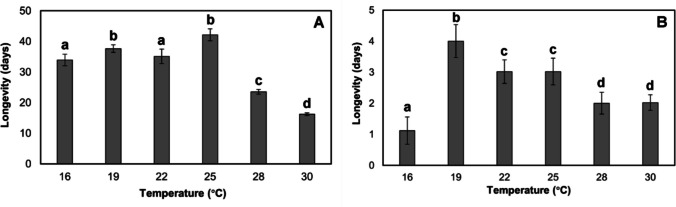
Mean (± SE) adult female longevity (days surviving) of *Prorops nasuta* (**A**) and *Phymastichus coffea* (**B**) at six constant temperatures. Different lowercase letters indicate significant differences according to Dunn’s test (*α* = 0.05)

Adult female longevity in *P. nasuta* was significantly different across temperatures (*χ*^2^ = 170.45, df = 5, *p* < 0.001). Longevity was similarly high for temperatures of 16–25 °C, with the highest mean longevity being 42 days at 25 °C. The maximum longevity recorded for a *P. nasuta* was 120 days after its emergence as an adult at 16 °C. Longevity decreased significantly at 28 °C and above; at the two highest temperatures tested (32 and 35 °C) all individuals died within several hours (Fig. 3A). Significant differences among temperatures were also observed for *P. coffea* longevity (*χ*^2^ = 260.34, df = 5, *p* < 0.001). At 32 and 35 °C, all individuals died after just 6 h. Mean adult longevity was lowest at 16 °C (1.12 ± 0.44 days) and highest at 19 °C (4.0 ± 0.53 days) (Fig. 3B). The maximum longevity recorded for a *P. coffea* wasp was six days at 19 °C.

**Fig. 4:**
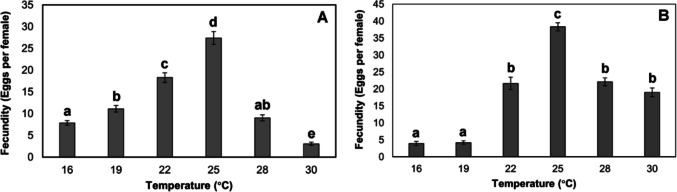
Mean (± SE) fecundity (eggs per female) of *Prorops nasuta* (**A**) and *Phymastichus coffea* (**B**) at six constant temperatures. Different letters indicate significant differences according to Dunn’s test (*α* = 0.05)

Fecundity in *P. nasuta* varied significantly among the temperatures examined (*χ*^2^ = 218.38, df = 5, *p* < 0.001). Fecundity was significantly higher at 25 °C (27 eggs/female) relative to all other temperatures, while the lowest fecundity was observed at 30 °C (3 eggs/female; Fig. 4A). Fecundity also varied significantly among temperatures for *P. coffea* (*χ*^2^ = 201.70, df = 5, *p* < 0.001), with the lowest average number of eggs laid at 16 °C and 19 °C (4 eggs/female), and the highest number of eggs laid at 25 °C (38 eggs/female) (Fig. 4B).

### Population Growth Rates

The life table for *P. nasuta* showed that the net reproductive rate (*R*_*0*_) increased with increasing temperature until reaching its highest level (16.6) at 25 °C (Table 2). The values of *R*_*0*_ observed at 16 °C and 30 °C were only 2.9 and 0.8 respectively, indicating a smaller population increase from one generation to another at these extreme temperatures. For the intrinsic rate of increase (*r*_*m*_), the highest value of 0.045 was observed at 25 °C, followed by 22 °C. The values for the finite rate of increase (*λ*) followed the same trend, with a peak of 1.05 at 25 °C. The values of *r*_*m*_ > 0 and λ > 1 indicate that between 16 and 28 °C, there is population growth. In contrast, at 30 °C, *λ* < 1, suggesting that this temperature is not favorable for population growth. The average time for one generation (*T*) ranged from 32.2 days at 30 °C to 90.4 days at 16 °C (Table 2).

**Table 2:** Estimated mean (95% CI) of parameters for the fertility life table of *Prorops nasuta* and *Phymastichus coffea* at constant temperatures. Different lowercase letters indicate significant differences among temperatures

Parameter	Temperature (°C)					
	16	19	22	25	28	30
*Prorops nasuta*						
* R*_*o*_	2.9 a (3.3–2.5)	4.6 b (5.3–3.9)	11.2 c (12.6–9.8)	16.6 d (18.3–14.9)	6.4 b (7.1–5.7)	0.8 e (1.0–0.6)
* T*	90.4 a (98.5–82.3)	72.3 b (78.8–65.8)	59.2 c (64.5–53.9)	41.1 d (44.8–37.4)	36.7 d (40.0–33.4)	32.2 d (35.4–29.3)
* λ*	1.01 a (1.02–1.01)	1.02 a (1.03–1.01)	1.03 ab (1.05–1.01)	1.05 b (1.06–1.04)	1.03 ab (1.05–1.01)	0.98 c (1.01–0.95)
* r*_*m*_	0.01 a (0.02–0.01)	0.02 ab (0.03–0.01)	0.03 ab (0.04–0.03)	0.05 b (0.05–0.04)	0.03 ab (0.04–0.02)	0.01 a (0.02–0)
*Phymastichus coffea*						
* R*_*o*_	–	2.6 a (7.6–0)	9.00 b (10.5–7.5)	14.5 c (20.1–8.9)	7.4 b (11.9–2.7)	5.8 b (8.9–2.7)
* T*	–	89.6 a (93.6–85.6)	46.1 b (51.7–41.1)	33.2 c (34.9–31.5)	27.1 d (28.7–25.5)	38.1 b (41.9–34.3)
* Λ*	–	1.00 a (1.02–0.98)	1.06 b (1.03–1.09)	1.08 b (1.13–1.03)	1.06 b (1.08–1.04)	1.05 b (1.06–1.04)
* r*_*m*_	–	0.003 a (0.02–0)	0.06 b (0.1–0.02)	0.09 b (0.12–0.06)	0.07 b (0.1–0.04)	0.05 b (0.07–0.03)

*R*_*0*_– net reproductive rate; *T* – generation time; λ –finite rate of increase; *r*_*m*_ – intrinsic rate of increase. The lower bound of the confidence interval resulted in negative values during bootstrapping and was corrected to zero to reflect the biological constraint that Net Reproductive Rate cannot be negative

For *P. coffea*, the net reproductive rate (*R*_*0*_) was highest (14.5) at 25 °C. The values of *R*_*0*_ observed at 19 and 30 °C were only 2.6 and 5.8, respectively, indicating a smaller population increase from one generation to the next at these extreme temperatures. The highest value for the intrinsic rate of increase (*r*_*m*_) was observed at 25 °C (0.09), followed by 28 °C (0.07) and 22 °C (0.06). According to these estimates, the temperature range of 22–28 °C is favorable for population growth of this parasitoid, with an optimum temperature at 25 °C (Table 2). The values obtained for the finite rate of increase (λ) followed the same trend, with a peak of 1.08 at 25 °C (Table 2). The values of *r*_*m*_ > 0 and λ > 1 indicate that between 19 and 30 °C there is population growth. The time for one generation (*T*) ranged from 27.1 days at 28 °C to 89.6 days at 19 °C (Table 2).

### Development Rate and Construction of a Mathematical Model

The linear model showed a good fit between the development time of *P. nasuta* at each of the life stages and temperature (*R*^2^ ≥ 0.94; Table 3). Complete development of the life cycle occurred within the range of 16–30 °C (Table 3). The larval stage had the lowest temperature threshold at 10.0 °C, and the pupal stage had the highest at 12.3 °C. On average, 366 degree-days were required to complete egg-to-adult development (Table 3).

**Table 3:** Linear regression of *Prorops nasuta* and *Phymastichus coffea* developmental rate at different life stages recorded over a range of constant temperatures (16–32 °C)

Developmental stage	Temperature range (°C)	Regression equation	*R*^2^	*t*	*K*
*Prorops nasuta*					
Egg	16–32	Y = − 0.2856 + 0.0220x	0.94	11.9	45
Larva	16–30	Y = − 0.0532 + 0.0054x	0.98	10.0	187
Pupa	16–30	Y = − 0.0925 + 0.0076x	0.94	12.3	133
Egg-adult	16–30	Y = − 0.0306 + 0.0027x	0.95	11.5	366
*Phymastichus coffea*					
Egg	16–32	Y = − 0.5222 + 0.0343x	0.93	15.0	29
Larva	19–32	Y = − 0.0949 + 0.0062x	0.99	15.3	160
Pupa	19–30	Y = − 0.0907 + 0.0063x	0.99	14.4	160
Egg-adult	19–30	Y = − 0.0440 + 0.0029x	0.99	14.9	344

*R*^2^ = coefficient of determination*; t* = minimum development threshold (°C); *K* = thermal constant (degree-days)

The linear model showed a good fit between the development time of *P. coffea* at each of the life stages and temperature (*R*^2^ ≥ 0.93) (Table 3). Complete development occurred at temperatures ranging from 19 to 30 °C. The pupal stage had the lowest temperature threshold at 14.4 °C, while the highest temperature threshold was observed at the larval stage (15.3 °C). On average, 344-degree days were required to complete the development from egg to adult (Table 3).

### Number of Generations in Colombia

**Fig. 5:**
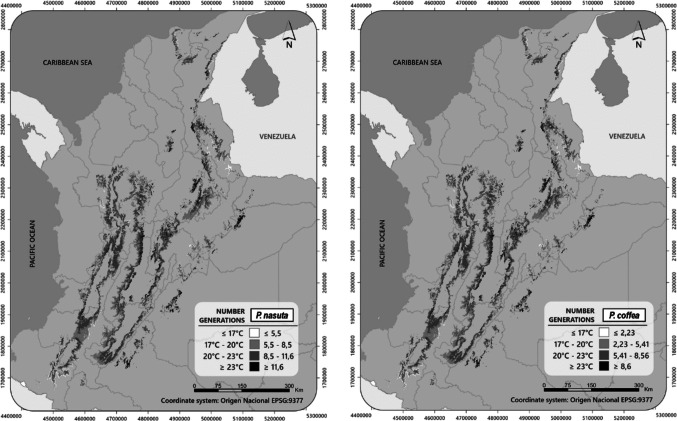
Number of generations per year of *Prorops nasuta* and *Phymastichus coffea* in Colombia, based on the 30-year average temperature normal

Based on the degree-days of four ranges of isotherms that represent the Colombian coffee region and are associated with the ranges of vulnerability of Colombian coffee to attack by *H. hampei* (Giraldo-Jaramillo et al. 2019), the number of *P. nasuta* generations per year (5.5–11.6) was estimated to be slightly higher than that estimated for *P. coffea* (2.2–8.6) (Table 4). The climate in Colombia is favorable all year round for these parasitoids, with the number of generations estimated to increase with increasing temperatures. However, these patterns are not always stable; variations from the normal climate in temperature and rainfall (El Niño and La Niña climatic events, which can affect the flowering time of coffee plants) can affect *H. hampei* populations, as well as populations of the parasitoids. The distribution of the potential number of generations shows that the coffee region in Colombia is suitable for both parasitoids (Fig. 5) and is a viable management option for controlling CBB.

**Table 4:** Potential number of generations of *Prorops nasuta* and *Phymastichus coffea* in four isotherms across the Colombian coffee region

	*P. nasuta* isotherms (°C)				*P. coffea* isotherms (°C)			
	≤ 17	> 17 and ≤ 20	> 20 and ≤ 23	> 23	≤ 17	> 17 and ≤ 20	> 20 and ≤ 23	> 23
January	0.47	0.47–0.72	0.72–0.97	0.97	0.19	0.19–0.46	0.46–0.73	0.73
February	0.42	0.42–0.65	0.65–0.88	0.88	0.17	0.17–0.42	0.42–0.66	0.66
March	0.47	0.47–0.72	0.72–0.97	0.97	0.19	0.19–0.46	0.46–0.73	0.73
April	0.45	0.45–0.70	0.70–0.94	0.94	0.18	0.18–0.44	0.44–0.71	0.71
May	0.47	0.47–0.72	0.72–0.97	1.01	0.19	0.19–0.46	0.46–0.73	0.73
June	0.45	0.45–0.70	0.70–0.94	0.94	0.18	0.18–0.44	0.44–0.71	0.71
July	0.47	0.47–0.72	0.72–0.97	1.01	0.19	0.19–0.46	0.46–0.73	0.73
August	0.47	0.47–0.72	0.72–0.97	1.01	0.19	0.19–0.46	0.46–0.73	0.73
September	0.45	0.45–0.70	0.70–0.94	0.94	0.18	0.18–0.44	0.44–0.71	0.71
October	0.47	0.47–0.72	0.72–0.97	0.97	0.19	0.19–0.46	0.46–0.73	0.73
November	0.45	0.45–0.70	0.70–0.94	0.94	0.18	0.18–0.44	0.44–0.71	0.71
December	0.47	0.47–0.72	0.72–0.97	0.97	0.19	0.19–0.46	0.46–0.73	0.73
Annual	≤ 5.51	5.51–8.49	8.49–11.43	≥ 11.55	≤ 2.23	2.23–5.41	5.41–8.59	≥ 8.59

## Discussion

We evaluated the development of the ectoparasitoid *Prorops nasuta* and the endoparasitoid *Phymastichus coffea* under eight constant temperatures in the laboratory. Our results suggest that *P. nasuta* can complete development over a period of 19–65 days at temperatures of 16–30 °C, while *P. coffea* can complete development over a period of 33–87 days at temperatures of 19–30 °C. In general, increasing temperatures resulted in faster development times, but temperatures above 30 °C were lethal to both parasitoids. Moderate temperatures of around 22–28 °C appeared to favor survival, longevity, and fecundity for both parasitoids.

Two earlier studies that examined development times at constant temperatures in *P. nasuta* found that development was completed more quickly than in the present study: De Toledo (1942) reported 23 days from egg to adult at 24 °C (vs. ~ 32 days in our study), while Abraham et al. (1990) estimated 22 days from egg to adult at 25 °C (vs. 29 days in our study). Klein-Koch et al. (1988) reported similar development times to our results, with 20–24 days from egg to adult at 27 °C. An earlier study on *P. coffea* development in the laboratory reported 35 days from egg to adult at 26 °C (Feldhege 1992), which is in line with our findings. Under natural field conditions, Espinoza et al. (2009) reported that the life cycle of *P. coffea* from egg to adult took an average of 47 days (mean temperature of 22 °C), and Vergara et al. (2001) reported 46 days (mean temperature of 23 °C). In contrast, Infante (2000) conducted a study on development and population growth rates in *P. nasuta* at five constant temperatures (18, 22, 27, 30, 35 °C) based on the climate in coffee-growing regions across Mexico. The author suggested that 27 °C was the optimum temperature for development and population growth of *P. nasuta,* which is in line with findings in the present study. There were also similarities with the present study in terms of fecundity trends and failure to complete development at extreme temperatures. However, there were some notable differences. Infante (2000) reported faster development times (2–12 days shorter) for each life stage, lower estimates for survival (69–76% at 22–27 °C), and highest longevity at a lower temperature (18 °C). The variation in the results reported among studies may be attributed to differences in rearing methodologies or genetic differences among the laboratory strains.

For successful biological control, Bernal and González (1993) suggested that a favorable relationship must exist between the developmental thresholds of parasitoids and their hosts. Generally, the lower temperature threshold of the parasitoid should be higher than that of the host (Campbell et al. 1974). This is to ensure that the parasitoids do not emerge before their hosts in the early season (Campbell and MacKauer 1975). In the present study, the lower developmental threshold for *P. nasuta* from egg-adult was 11.5 °C and the thermal constant was 366 DD, which is in line with earlier studies conducted by Infante (2000) and Benassi and Bussoli (2009). For *P. coffea*, the lower developmental threshold was higher at 14.9 °C and the thermal constant was 344 days from egg-adult. The reported lower developmental threshold for *H. hampei* is 11.25 °C (Costa and Villacorta 1989), which is lower than that reported for *P. nasuta* and *P. coffea,* confirming that *H. hampei* is a suitable host for these parasitoids as it can survive and begin development at a lower temperature. According to Infante et al. (2001b), the bethylid wasps can also parasitize other internal feeding Coleoptera and certain Lepidoptera. Further research is needed to determine if *P. nasuta* utilizes alternative hosts for survival in the Colombian coffee agroecosystem.

For parasitoids to be effective in regulating pest numbers, they should have good host finding and exploitation potential, have high longevity, and high fecundity. However, extremes of temperature may offset these attributes, with negative consequences on biological control. Exposure of parasitoids to extreme temperatures has been reported to decrease adult longevity (Pandey and Johnson 2005; Foerster and Doetzer 2006), mating success, and the proportion of females (Augustin et al. 2022), as well as fecundity (Levie et al. 2005; Pandey and Johnson 2005). In the present study, the development of *P. nasuta* and *P. coffea* was negatively affected by the upper and lower extremes of the temperature ranges examined. At the lowest temperature of 16 °C, *P. coffea* was unable to complete development, and at 32 and 35 °C, both parasitoids were unable to complete development. Survival, longevity, and fecundity were also significantly reduced at the lower and upper temperatures examined. These results are in line with the study by Infante (2000), who reported high mortality of immature stages and reduced adult longevity at 30 and 35 °C. Higher longevity at cooler temperatures may be related to a reduction in metabolic processes (Lasota and Kok 1986). This is important since longer parasitoid lifespans increase the possibility of successful host parasitism. However, the naturally short longevity of *P. coffea* (typically only a few days) is characteristic of koinobiont endoparasitoids that emerge with limited energy reserves and rarely feed as adults, making rapid host attack essential. It is also noteworthy that CBB development and fecundity are inhibited at extreme temperatures (Giraldo-Jaramillo et al. 2018), further supporting that the host-parasitoid biology of these two insects exhibits consistent responses to environmental factors such as temperature.

We also found that the sex ratio in *P. nasuta* was highly skewed at extreme temperatures, with warmer temperatures favoring males and cooler temperatures favoring females. An earlier study examining the sex ratio of *P. nasuta* reported a male proportion of 0.21 at 18 °C (Infante et al. 2005), which is in line with our results. Other studies on parasitoids have reported that at high temperatures the sex ratio shifts towards a male bias (Power et al. 2022; Tabebordbar et al. 2022; Bressac et al. 2023), due to a combination of reduced male fertility and ovipositing females laying fewer fertilized eggs. In contrast, there was no difference in sex ratio across temperatures for *P. coffea*. The differences observed in sex ratio between these two parasitoids may be related to the nature of host parasitism and development. *Prorops nasuta* eggs develop externally on the juvenile host compared to *P. coffea* that lays its eggs internally in the adult host, where they are likely more insulated from varying temperatures.

According to Giraldo-Jaramillo et al. (2019), areas with temperatures > 21 °C are considered to have moderate to high susceptibility to *H. hampei*. Our results suggest that these same areas will be favorable for *P. nasuta* and *P. coffea*. However, temperature increases associated with climate change may decrease survivorship for the immature stages and thus decrease the efficacy of these parasitoids as a biocontrol (Chidawanyika et al. 2019). Climate warming among interacting trophic levels has been reported as a catalyst for extinction in species at higher trophic levels, such as parasitoids (Jones 2008; Hance et al. 2007; Northfield and Ives 2013; Mellard et al. 2015) with population sizes mediating the evolutionary dynamics (Oostra et al. 2018). High temperatures associated with climate change may affect the host or parasitoid directly or indirectly through temperature-mediated species interactions (Péré et al. 2013; Malinski et al. 2023); predicted changes in ecological outcomes due to climate change may be mitigated by thermal plasticity or acclimation in the parasitoid (Ramos Aguila et al. 2023). Understanding the ideal temperature ranges for development and establishment of parasitoids will allow planning for future climate scenarios and can help to predict the adaptive capacity that these species will have in the Colombian coffee agroecosystem.

Our findings can be used to predict where *P. nasuta* and *P. coffea* would be most successful in establishing in Colombia. Colombian coffee is cultivated between 1 and 11° north latitude, with around 93% of the total planted area between 1 and 8° N (Jaramillo 2018). Given that Colombia is in the tropics, the thermal conditions only vary slightly across the coffee-growing region (1 °C per 1000 km of latitudinal arc), with flowering and fruiting occurring year-round (Arcila-Pulgarin et al. 1993). Air temperature only differs in relation to altitude. For the coffee-producing region, with every increase of 100 m in elevation there is a decrease of 0.61 °C, with little monthly variability at the same altitude (Jaramillo 2018). According to Maldonado and Benavides (2007), *P. nasuta* was recovered in Colombian growing regions from 1100 m (~ 23 °C) to 1850 m (~ 17 °C). Our findings suggest that the potential number of generations per year for *P. nasuta* in Colombia fluctuates between 5.5 and 11.6, with optimal developmental ranges between 22 and 25 °C, and optimal ranges for establishment occurring between 19 and 28 °C. For *P. coffea*, we found that the number of generations per year in Colombia is slightly lower at 2.2–8.6, with optimal ranges for development between 22 and 25 °C, and optimal ranges for establishment occurring between 19 and 28 °C.

The success of parasitoid biocontrol agents depends on host density and the timing of seasonal activities (Berryman 1999; Jeffs and Lewis 2013). Although *H. hampei* and their parasitoids share similar thermal niches, population growth is very different for these species. The net reproductive rates (*R*_0_) of *H. hampei* can be up to eight times higher than that calculated for *P. nasuta* and *P. coffea*, which suggests that parasitoids alone are not enough to suppress *H. hampei* populations in the field. However, when incorporated with other IPM strategies, the use of parasitoids at specific times can help reduce *H. hampei* infestation. A *H. hampei* control strategy recently proposed by Benavides et al. (2023) involves multiple releases of *P. nasuta* months before the renewal of coffee plantations is carried out (annually 20% of the planted area in Colombia is stumped). During stumping, the physical disturbance and absence of coffee berries cause the *H. hampei* to move from areas in which populations have had at least 5 years of continuous cycles to build up in size (Castaño et al. 2005). This initial release of *P. nasuta* to lower *H. hampei* populations would be followed by releases of *P. coffea* into nearby productive fields with coffee berries that may be colonized by the dispersing *H. hampei* (Benavides et al. 2023). In this way, the release of two different parasitoids may complement one another to more successfully control *H. hampei* in coffee fields.

To thrive, *P. nasuta* and *P. coffea* need a consistent supply of hosts. Therefore, when releases are carried out in the field, there must be a large existing *H. hampei* population to avoid the migration of the parasitoids to other regions surrounding the coffee plantations. Bethylid parasitoids can increase significantly in abandoned coffee plots where the berries are not harvested and may be heavily infested by *H. hampei* (Jaramillo et al. 2006). Given that these abandoned plots do not receive any *H. hampei* management, conditions would be favorable for the development and population growth of *P. nasuta* since to initiate egg laying, there must initially be numerous *H. hampei* eggs or larvae available. It is therefore possible that these abandoned plots may serve as a reservoir where *P. nasuta* populations increase. Abandoned fields may also serve as a source of *P. coffea* to invade commercial coffee fields with growing *H. hampei* populations and allow *P. coffea* populations to survive when surrounding fields are stumped.

## Conclusions

We found that the optimal temperature for mass-rearing *P. nasuta* and *P. coffea* (based on development and population growth) was 25 °C, while for field releases the optimal temperature range is between 22 and 28 °C. Our results suggest that the Colombian coffee region should support the establishment of *P. nasuta* and *P. coffea* since the thermal conditions vary little throughout the year, allowing a constant supply of coffee and *H. hampei*. Under Colombian growing conditions, we expect that between 5.5–11.6 generations of *P. nasuta* and 2.2–8.6 generations of *P. coffea* may be produced annually. Future studies should examine the development, fecundity, and survival of these parasitoids under field conditions to further assess these life history characteristics under naturally fluctuating temperatures.

## Data Availability

Data is available in the USDA National Agriculture Library Ag Data Commons.
